# Diagnostic utility of IBEX Bone Health for assessment of osteoporosis from knee radiographs

**DOI:** 10.3389/fmolb.2025.1671053

**Published:** 2025-12-10

**Authors:** Robert Meertens, Ben Lopez, Mike Gundry, Ben Crone, Wavell Vigers, Michelle Evans, Richard McWilliam, Sarah Jarvis

**Affiliations:** 1 Department of Health and Care Professions, University of Exeter, Exeter, United Kingdom; 2 Ibex Innovations Ltd., Sedgefield, United Kingdom

**Keywords:** osteoporosis, bone mineral density, knee radiographs, distal femur, proximal tibia, opportunistic screening, artificial intelligence

## Abstract

**Purpose:**

This study reports testing of IBEX Bone Health (IBEX BH) software, applied following acquisition of knee digital radiographs (DRs). IBEX BH analyses the DR to measure areal bone mineral density (aBMD) and T-scores. This study investigates aBMD and T-score at the knee as a biomarker to identify patients at high risk of osteoporosis.

**Methods:**

A 497-participant single-centre, non-randomised, prospective study was carried out comparing a) IBEX BH, a quantitative digital radiography software device, to b) Dual-energy X-ray Absorptiometry (DXA). Participants underwent left and right knee DRs, and knee, pelvis and lumbar spine DXA scans. Analysis evaluates the efficacy of IBEX BH as a knee densitometry device, comparing performance to that of reference standard DXA at the same anatomical site. It further evaluates the area under the receiver operating characteristics curve (AUC) performance of IBEX BH in predicting osteoporosis, defined by clinical reports from hip and/or spine DXA. This is compared against DXA referral recommendations derived from National Osteoporosis Guideline Group (NOGG) guidelines, which use 10-year fracture risk calculated by the fracture risk assessment tool (FRAX) without neck of femur (NoF) aBMD.

**Results:**

Correlations between IBEX BH aBMD and DXA aBMD at the same anatomical sites were 0.91 (95% confidence interval (CI) [0.90, 0.93]) for the proximal tibia (PT) and 0.87 (95%CI [0.84, 0.89]) for the distal femur (DF). For the PT and DF, the AUC for IBEX BH for discriminating central osteoporosis was 0.91 (95%CI [0.87, 0.95]) and 0.88 (95%CI [0.82, 0.93]) respectively, compared to 0.93 (95%CI [0.9, 0.97]) and 0.92 (95%CI [0.88, 0.96]) for DXA. In operating point analysis, the sensitivity and specificity to central osteoporosis of PT IBEX BH T-score was 0.66 95%CI [0.50, 0.78] and 0.94 95%CI [0.89, 0.96] respectively, compared to a sensitivity and specificity of 0.66 95%CI [0.50, 0.79] and 0.69 95%CI [0.63, 0.75] respectively for NOGG referral thresholds based on FRAX without NoF aBMD.

**Conclusion:**

Ibex BH shows strong correlation to DXA at the same anatomical site, and superior ability to FRAX without NoF aBMD for classifying central osteoporosis. Results demonstrate the potential utility of IBEX BH as a densitometer and opportunistic osteoporosis screening tool.

## Introduction

Osteoporosis is a systemic skeletal disease characterized by reduced bone mass and microarchitectural deterioration, leading to increased fragility and fracture risk (WHO Scientific Group on the Prevention and Management of Osteoporosis, 2003; National Osteoporosis Guideline Group UK NOGG, 2024). Osteoporosis remains a pervasive global health issue, particularly among aging populations, due to its high prevalence and the significant morbidity associated with fragility fractures (Salari et al., 2021). The World Health Organization defines osteoporosis as an areal bone mineral density (aBMD) 2.5 standard deviations or more below the young adult mean at the Neck of Femur (NoF) (Hernlund et al., 2013). Despite advancements in diagnostic tools and treatment options, underdiagnosis and undertreatment persist (Hernlund et al., 2013; Kanis et al., 2021). In the United Kingdom, for instance, approximately 66% of women who meet treatment criteria for osteoporosis remain untreated, underscoring a substantial treatment gap (National Osteoporosis Guideline Group UK NOGG, 2024). This is particularly significant given that hip and femoral fractures represent the most costly and debilitating consequences of osteoporosis. Hip fractures were responsible for more unplanned hospital admission bed days than any other condition apart from pneumonia in 2022/23 with 1-year mortality rates as high as 30% following a hip fracture (Hernlund et al., 2013; NHS England, 2023).

Dual-energy X-ray absorptiometry (DXA) is the clinical reference standard for aBMD measurement and fracture risk assessment (WHO Scientific Group on the Prevention and Management of Osteoporosis, 2003; National Osteoporosis Guideline Group UK NOGG, 2024), typically used clinically in conjunction with the Fracture Risk Assessment Tool (FRAX) to aid clinical decision making on treatment (Centre for Metabolic Bone Diseases UoS, 2023). However, DXA has inherent limitations as a diagnostic tool (Choksi et al., 2018) and access to DXA scanning is limited in many healthcare settings, often leading to delayed diagnosis until after a fracture has occurred. For example, in the United Kingdom two of three patients wait over 3 months for a DXA scan (Royal College of Physicians, 2025). In 1 in 5 cases, people breaking a bone sustain 3 or more fractures before receiving a diagnosis (Kanis et al., 2021). This reactive approach diminishes the opportunity for early intervention, which is crucial for reducing fracture incidence and associated healthcare costs (Kanis et al., 2021).

As evidence suggests subjective interpretation of radiographs for radiographic osteopenic appearances is variable in performance and execution (Vigers et al., 2025), opportunistic screening has emerged as a promising strategy to bridge this treatment gap by improving case finding even before primary fracture. By leveraging existing radiographic imaging obtained for other clinical indications, healthcare providers can assess bone health without necessitating additional referrals or imaging studies (Chaisen et al., 2024; Mousavi et al., 2025). In particular, the IBEX Bone Health (IBEX BH) software applies inverse modelling to standard digital radiographs to estimate the easily codable aBMD and T-score post-acquisition (Lopez et al., 2024; Meertens et al., 2024; Söreskog et al., 2025). The software provides a secondary Digital Imaging and Communications in Medicine (DICOM) capture of results, allowing unobtrusive access to results, and objective data to support clinical action. Previous research has demonstrated the feasibility of estimating aBMD from wrist radiographs using an image-based software application (Lopez et al., 2024; Meertens et al., 2024) and it has been shown that implementation of opportunistic screening has the potential to be cost-saving (Söreskog et al., 2025).

Given the absence of a systematic screening programme, the high incidence of osteoporosis in over 50s, and the high frequency of knee radiographs in this age group, there is a clear imperative for objective automated opportunistic screening at the knee, which remains relatively underexplored. (National Health Service UK, 2023). The proximal tibia (PT) and distal femur (DF) are frequently imaged during routine evaluations for knee pain, trauma, and preoperative planning for orthopaedic procedures. Low bone mineral density identified prior to knee replacement may also influence post-operative management for patients (Chang et al., 2023). Given the high morbidity and mortality associated with hip and knee fractures, early identification of osteoporosis in these regions could significantly impact patient outcomes (Elmahdy and Sebro, 2023; Sarhan et al., 2024).

This study aims to evaluate the efficacy of the IBEX BH software for densitometry and opportunistic assessment of bone health using knee radiographs, specifically focusing on the PT and DF. The accuracy of IBEX BH in measuring aBMD at the PT and DF regions will be assessed. Furthermore, the predictive capability of aBMD at these regions for central osteoporosis will be determined. The prediction of central osteoporosis will finally be compared against National Osteoporosis Guideline Group (NOGG) intervention guidelines, which utilise FRAX (without NoF aBMD) as a method for identifying patients who are at high risk of osteoporosis and therefore require a DXA scan.

## Materials and methods

### Study design and participants

A prospective, single-centre study was conducted with volunteer participants. Participants (n = 497) included 241 adults aged ≥50 years (range [50,89]) (intended use population). A healthy-normal reference population was also required for the calculation of a T-score, due to the absence of publicly available data (Cirnigliaro et al., 2022). Healthy volunteers aged 20–30 (n = 256) were also recruited, subject to exclusion criteria.

The exclusion criteria were inappropriate age, the inability to consent, past history of bilateral knee or hip fractures/implants, or pregnancy. Where patients otherwise met inclusion criteria, participants with unilateral total replacement, implants or fixation in any of the anatomical regions imaged had that anatomy omitted from the study. Ethical approval was obtained through the UK Health Research Authority (REF 23/YH/0089).

### Imaging and software application

Participants underwent anterior-posterior (AP) digital radiographs of the left and right knee using an AGFA DR100 x-ray system, with standardized imaging parameters (60 kVp, 5 mAs, no grid, source to image distance (SID) 115 cm). DR exposures were each carried out by one of four clinically qualified radiographers. The IBEX BH software was calibrated to the DR system at the beginning of the study using a set of manufacturer-provided phantoms, passing all quality assurance criteria.

During the same participant visit, DXA scans assessing aBMD at the PT and DF were performed alongside bilateral hip and lumbar spine (LS) DXA acquisitions using a GE Lunar system. Precision and accuracy were monitored daily using a manufacturer-supplied phantom. All quality assurance checks were within the manufacturer’s tolerances throughout the study. A team of five clinically qualified radiographers carried out the DXA measurements. All DXA images were reported by a qualified DXA reporting radiographer; a Professor in Musculoskeletal Imaging. Where appropriate, regions of interest (ROIs) were manually repositioned to correct for errors in DXA’s automated ROI selection algorithm.

Radiographs were post-processed with IBEX BH to extract bone density maps and calculate aBMD at the PT and DF. The software assesses the raw data received by the image receptor and applies an inverse modelling algorithm to determine the contribution of bone to the image based on the scattering properties of tissue, thus formulating a marker of bone density that can be mapped at the pixel level. The principles behind IBEX BH software are previously described in detail (Lopez et al., 2024; Meertens et al., 2024).

**FIGURE 1:**
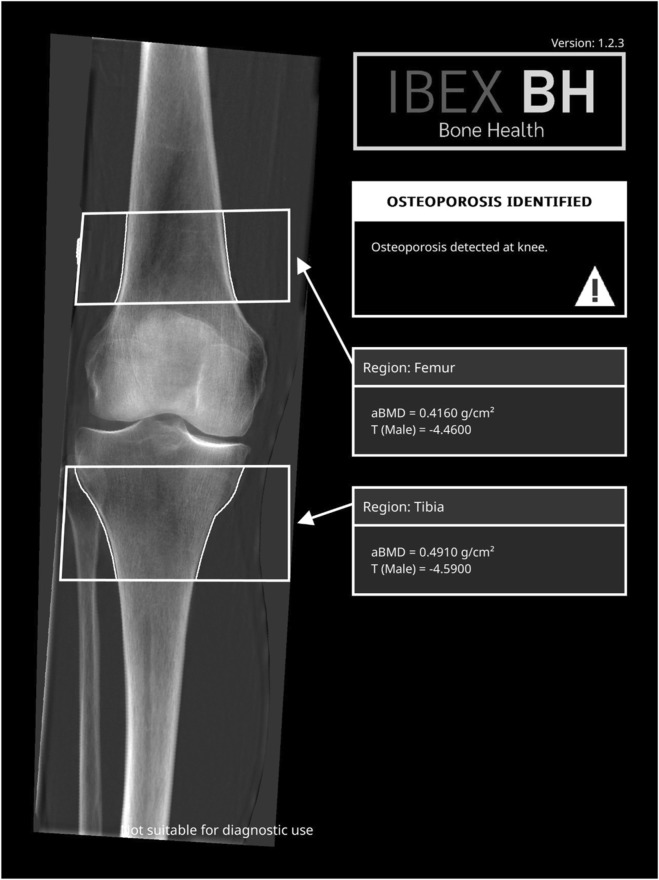
Example report from the IBEX BH software. Left: bone density image with ROIs and segmentations visible for manual inspection by reporting clinician. Right: aBMD and T-scores for both ROIs, and a clear indication that the individual is high risk.

An example IBEX BH report for the knee containing the bone density image, the ROIs, aBMD and T-scores is shown in Figure 1.

### Regions of interest at the knee

**FIGURE 2:**
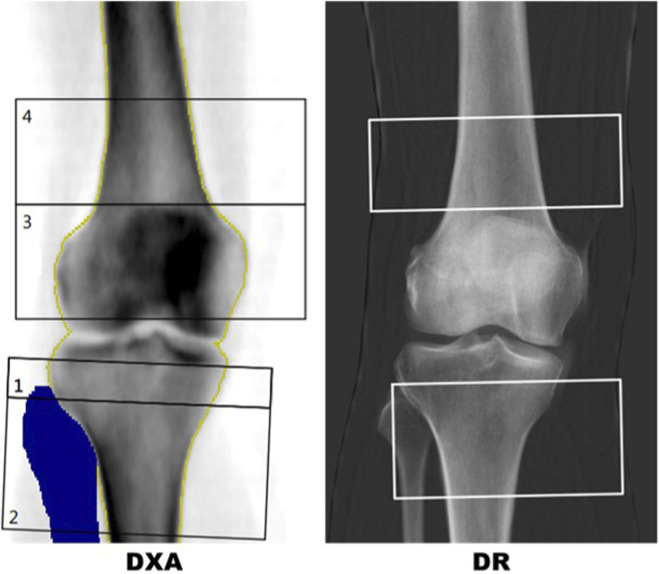
Left: DXA report with template for distal femur (box 4) and proximal tibia (box 2). The blue region is a mask for the fibula showing the region that has been removed from the proximal tibia region of interest (ROI). Right: IBEX BH bone density image with semi-automated distal femur and proximal tibia boxes.

DXA analysis at the knee utilised a bespoke template for ROI placements (see Figure 2) designed to avoid superimposition from the patella and any sclerotic changes relating to the tibial joint surface. Two ROIs for measurement of aBMD and T-score were defined for the AP knee DXA scans. The PT ROI was defined as a 50 × 100 mm rectangle centred 40 mm in the inferior direction from the midpoint of the tibial plateau. The fibula bone was removed from the DXA analysis. The DF ROI was defined as a 40 × 110 mm rectangle centred some distance superior to the intercondylar fossa. This distance varied per scan and was defined as the height of the patella. Three blinded researchers carried out an inter-operator precision exercise for knee DXA, post-processing 10 cases, three times each, resulting in a Root Mean Square Coefficient of Variation of 1.39% for PT and 1.18% for DF analysis of aBMD.

In DR scans, segmentation masks of the femur, tibia, fibula and patella were used to replicate the DXA procedure in a semi-automated approach. The masks were manually labelled by a team of labellers but an automated machine vision algorithm was used to identify the ROIs using the segmentation of the femur, tibia, fibula and patella. A comparison of typical DR and DXA ROIs for the same patient are shown in Figure 2.

### Questionnaires and clinical data collection

All participants completed FRAX (Centre for Metabolic Bone Diseases UoS, 2023) questionnaires to provide 10-year hip and major fracture risk estimates, calculated both with and without femoral neck aBMD values obtained from DXA scans. FRAX is a clinically adopted fracture risk assessment tool designed to assist in osteoporosis treatment decisions based on clinical risk factors for fragility fracture. FRAX without aBMD scores were input into the NOGG guidelines intervention graph to identify those participants for whom a DXA scan would be recommended and those identified as being at lower risk, who would be given lifestyle advice alone in a clinical scenario. FRAX with femoral neck aBMD were calculated to provide the clinical standard for treatment decision making based on fragility fracture risk. The minimum age for FRAX is 40 so FRAX outputs are not available for the healthy-normal cohort.

### Comparators and outcomes

Primary outcomes from analysis of aBMD and T-score at the PT and DF are (i) the aBMD by IBEX BH and DXA and (ii) central osteoporosis (T-score ≤ −2.5 at the NoF or LS). The prediction of central osteoporosis is compared to FRAX without aBMD (Centre for Metabolic Bone Diseases UoS, 2023) and NOGG referral criteria (National Osteoporosis Guideline Group UK NOGG, 2024).

The secondary outcomes is treatment recommendation after FRAX with femoral neck aBMD based on NOGG treatment guidelines. The ability of IBEX BH and all FRAX clinical risk factors (CRFs) excluding NoF aBMD to predict this outcome is compared to FRAX without aBMD results.

To calculate T-scores for the PT and DF ROIs, the healthy-normal DXA knee data was used to estimate the population mean and standard deviation for the healthy-normal cohort. The ROI and sex specific knee T-scores were then calculated using the following formula

$$T=\frac{aBMD-\mu }{\sigma }$$

where μ is the sex and ROI specific estimate of the population mean and σ is the sex and ROI specific estimate of the population standard deviation. The manufacturer T-scores were used for the LS and NoF. The minimum value of left and right NoF aBMD was used as an input to the FRAX model.

### Statistical analysis

To describe the continuous demographic and DXA bone health variables, the mean and standard deviation are reported. To describe the categorical demographic and DXA classifications, the percentage in that category are reported. The analysis is performed patient-wise for the healthy-normal and intended use cohorts separately.

To analyse IBEX BH as a tool to measure aBMD at the PT and DF, i) the correlation, ii) mean difference between IBEX BH and DXA aBMD and iii) the standard deviation of that difference are reported. To analyse IBEX BH as a tool to discriminate T-score ≤ −2.5 and T-score ≤ −1 at the PT and DF, the AUCs are reported. The analysis is performed image wise for the healthy-normal and intended use cohorts separately, and for Females and Males for the intended use cohort.

The correlation between PT and DF T-scores, and NoF and LS T-scores are reported. Where a left and right measurement is available for the same patient, the minimum T-score of left and right is used to align with clinical practice for reporting DXA scans of the hips.

To analyse IBEX BH as a tool to identify patients at high risk of central osteoporosis, a logistic regression model is fitted with age as an additional risk factor. Age is added because Z-scores are often better aligned than T-scores when comparing peripheral and central DXA sites. The AUC for central (minimum of NoF and LS T-score) NoF and LS osteoporosis and osteopenia are reported. The analysis is performed for the intended use population, and for Males and Females separately.

To analyse the comparator FRAX without femoral neck aBMD, three possible implementations of FRAX as a central osteoporosis screening tool are assessed. The three methods are i) setting a threshold on 10 years hip fracture risk (Shepstone et al., 2018), ii) setting a referral threshold on major osteoporotic fracture risk and iii) using the NOGG guidelines. For the first two implementations, the AUC for central osteoporosis or osteopenia are reported. For the third implementation, if the FRAX without aBMD fracture risk is above the “measure aBMD” referral line on the intervention graph, the participants are considered high-risk. The sensitivity and specificity of IBEX BH are reported for two intervention thresholds: one which matches NOGG guidelines sensitivity and one which matches the specificity.

To assess how well knee aBMD can be used as a tool to identify patients who will be recommended treatment by FRAX with aBMD and NOGG guidelines, knee T-scores are integrated with FRAX clinical risk factors in a risk prediction model. Logistic regression models are fit using CRFs (age, sex BMI, previous fracture, parental hip fracture, smoker, glucocorticoids, rheumatoid arthritis, secondary osteoporosis, >3 units alcohol) and a knee T-score. For prediction of the output of a fracture risk model, all CRFs are required as they include independent information for fracture risk prediction. Forward backward model selection is performed to remove any variables that do not significantly impact the risk prediction model’s performanceThe analysis is performed patient-wise for the intended use cohort. The AUC for FRAX without aBMD is also calculated for comparison.

The amount of missing data was small. Therefore, in the event of missing data, participant records were removed from the corresponding part of the analysis and no imputation was performed. All statistics are reported with their corresponding 95% confidence intervals (CIs), which by convention corresponds to a predetermined significance level of 0.05. The statistical analysis was performed with the statistical software package, R (R Core Team R. R, 2013) version4.0.1.

### Sample size statement

The primary aim of this study was to evaluate the ability of IBEX BH, when measuring aBMD at the DF and PT ROIs, to identify patients with a central DXA T-score ≤−2.5, aiming for an AUC greater than 0.79 achieved by quantitative ultrasound (QUS) (Nayak et al., 2006). For this objective, a total of 162 participants were required to achieve 0.8 power and a 0.05 significance level, based on a prevalence of 0.18 and an effect size of 0.14. Consequently, the study is sufficiently powered to meet its primary objective.

The aim of the healthy-normal data collection was to estimate the population mean and variance of aBMD in each ROI on the knee. Assuming the population standard deviation is 0.036 (Meertens et al., 2024), 134 males and females are required to estimate the sample mean of the knee ROI at 0.5% accuracy. Therefore, the study has sufficient participants to estimate the health-normal population mean with under 1% error.

## Results

Results are presented for 497 participants, 256 (122m/134f) from the healthy-normal cohort and 241 (114m/127f) from the intended use cohort. All participants had at least one DR and DXA knee scan available. In total there were 991 DR knees analysed and 990 DXA knees (one DXA knee was not reported due to poor positioning). For the intended use cohort, all participants had at least one valid DXA hip result. Nine did not have a valid DXA spine result. For the healthy-normal cohort, 1 participant did not have their hip DXA reported and 3 did not have their LS reported. Table 1 contains the summary statistics for the continuous demographic variables and Table 2 contains the summary statistics for the categorical demographic variables.

**TABLE 1:** Summary statistics for the continuous demographic, DXA and FRAX variables. Each cell contains the mean (standard deviation) for the variable. Each column is for a different continuous variable. Each row is the two cohorts, health normal (Age ≤30, n = 256) and intended use (Age >50, n = 241) populations.

	Age	BMI	Femur aBMD	Femur T-score	Tibia aBMD	Tibia T-score	NoF T-score	Spine T-score	FRAX major	FRAX hip
Age ≤30	23.77 (3.35)	24.43 (4.52)	1.14 (0.17)	−0.12 (0.98)	1.28 (0.19)	−0.12 (0.98)	0.12 (1.12)	0.07 (1.12)	NA (NA)	NA (NA)
Age >50	65.4 (9)	25.94 (4.77)	1.04 (0.2)	−0.75 (1.14)	1.14 (0.22)	−0.89 (1.13)	−1.05 (1.12)	−0.38 (1.62)	7.47 (4.86)	1.65 (2.18)

**TABLE 2:** Summary statistics for the categorical demographic, DXA and FRAX variables. Each cell contains the percentage of the participants who are in the category in the column title. Each row is the two cohorts, health normal (Age ≤30, n = 256) and intended use (Age >50, n = 241) populations.

Serial number	Sex (Female)	Parental hip fracture (yes)	Previous fracture (yes)	Smoker (yes)	Rheumatoid arthritis (yes)	Secondary osteoporosis (yes)	High alcohol use (Yes)	Knee femur T-score ≤ −2.5	Knee tibia T-score ≤ −2.5	
Age ≤30	52.34	0.39	0	7.42	0.78	0.39	2.34	0.78	0.39	
Age >50	52.7	11.2	7.88	1.24	2.9	1.24	10.37	5.81	9.13	

	NoF T-score ≤ −2.5	Spine T-score ≤ −2.5	Central T-score ≤ −2.5	Knee femur T-score ≤ −1	Knee tibia T-score ≤ −1	NoF T-score ≤ −1	Spine T-score ≤ −1	Central T-score ≤ −1	NOGG high risk	NOGG very high risk
Age ≤30	0.39	0.4	0.8	19.14	19.92	16.08	20.63	20.63	NA	NA
Age >50	10.37	9.48	16.24	37.76	39	55.6	37.07	39.74	7.47	3.32

**FIGURE 3:**
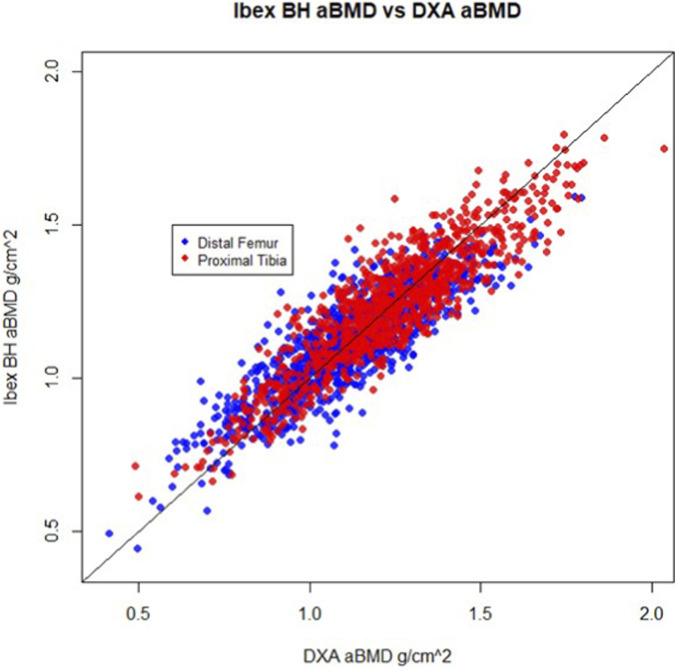
Scatter plot showing DXA aBMD against IBEX BH aBMD at the distal femur (blue) and proximal tibia (Red) regions of interest for both the intended use and healthy-normal cohorts. For the intended use cohort, the correlation between IBEX BH and DXA at the DF is 0.87 (95% CI [0.84, 0.89]) and at the PT 0.91 (95% CI [0.90, 0.93]). For the healthy normal cohort, the correlation between IBEX BH and DXA at the distal femur is 0.82 (95% CI [0.79, 0.85]) and at the proximal tibia 0.85 (95% CI [0.82, 0.87]).

Table 3 contains accuracy metrics for comparison of IBEX BH at the knee to DXA at the knee and Figure 3 shows the correlation plots of IBEX BH vs. DXA. For the intended use cohort, IBEX BH estimates showed strong correlation with DXA-derived aBMD measurements at the PT (r = 0.91 (95% CI [0.90, 0.93])) and DF (r = 0.87 (95% CI [0.84, 0.89])). ROC analysis demonstrated high discrimination for T-score ≤ −2.5 at the knee by DXA: (0.98 95% CI [0.97, 0.99] for the PT and AUC = 0.98 (95% CI [0.96, 1.00]) for the DF.

**TABLE 3:** Performance metrics comparing IBEX BH and DXA at the knee. The comparisons are split for each ROI and the two cohorts. Each cell contains the statistic and a 95% confidence interval. The comparison is performed per image, n = 990. Data for subgroups of males and females >50 are also presented (female n = 255; male n = 228).

	Correlation aBMD	Mean difference aBMD	Standard deviation difference aBMD	AUC T-score ≤ - 2.5	AUC T-score ≤ −1
Femur age ≤30	0.82 [0.79, 0.85]	0.01 [0, 0.02]	0.1 [0.09, 0.1]	0.98 [0.95, 1]	0.91 [0.88, 0.94]
Femur age >50	0.87 [0.84, 0.89]	−0.01 [-0.02, −0.01]	0.1 [0.1, 0.11]	0.98 [0.96, 1]	0.93 [0.9, 0.95]
Tibia age ≤30	0.85 [0.82, 0.87]	0.00 [-0.01, 0.00]	0.1 [0.1, 0.11]	0.99 [0.98, 1]	0.91 [0.88, 0.94]
Tibia age >50	0.91 [0.9, 0.93]	0.00 [0.00, 0.01]	0.09 [0.09, 0.1]	0.98 [0.97, 0.99]	0.97 [0.95, 0.98]
Femur age >50(Female)	0.86 [0.82, 0.88]	0 [-0.01, 0.01]	0.09 [0.09, 0.1]	0.95 [0.92, 0.99]	0.94 [0.92, 0.97]
Tibia age >50 (female)	0.92 [0.9, 0.94]	0.03 [0.01, 0.04]	0.08 [0.07, 0.09]	0.96 [0.94, 0.98]	0.97 [0.96, 0.99]
Femur age >50 (male)	0.8 [0.75, 0.85]	−0.03 [-0.05, −0.02]	0.11 [0.1, 0.12]	1 [1,1]	0.91 [0.87, 0.95]
Tibia age >50 (male)	0.89 [0.86, 0.92]	−0.02 [-0.03, 0]	0.1 [0.09, 0.1]	1 [1,1]	0.96 [0.94, 0.98]

For the intended use cohort, the correlation between IBEX BH T-score and NoF T-score is 0.68 (95% CI [0.61, 0.74]) and 0.65 (95% CI [0.57, 0.72]) for the PT and DF ROIs respectively. For DXA PT and DF ROIs, these correlations are 0.73 (95% CI [0.66, 0.78]) and 0.70 (95% CI [0.64, 0.76]).

Table 4 contains accuracy metrics for the comparison of IBEX BH at the knee, DXA at the knee and FRAX without femoral neck aBMD for detecting osteoporosis at the NOF and/or LS.

**TABLE 4:** Performance metrics comparing knee T-scores and FRAX 10-year fracture risk as a predictor of osteoporosis for the intended use population n = 241 for central and Neck of Femur and n = 232 for lumbar spine. Each cell contains the area under the receiver operating characteristic curve and a 95% confidence interval. Osteoporosis at a site is defined by a T-score ≤ −2.5 at that site and osteopenia a T-score ≤ −1. Central Osteoporosis/osteopenia is defined by participants that have a T-score ≤ −2.5/-1 at either the Neck of Femur or Lumbar spine.

	Central osteoporosis	Central osteopenia	Neck of femur osteoporosis	Neck of femur osteopenia	Lumbar spine osteoporosis	Lumbar spine osteopenia
All						
Age	0.76 [0.68, 0.84]	0.71 [0.64, 0.78]	0.69 [0.59, 0.79]	0.72 [0.65, 0.78]	0.85 [0.79, 0.91]	0.67 [0.6, 0.74]
Age + ibex BH femur	0.88 [0.82, 0.93]	0.84 [0.8, 0.89]	0.84 [0.77, 0.9]	0.86 [0.81, 0.9]	0.94 [0.9, 0.98]	0.77 [0.71, 0.84]
Age + ibex BH tibia	0.91 [0.87, 0.95]	0.89 [0.84, 0.93]	0.88 [0.83, 0.94]	0.87 [0.83, 0.92]	0.94 [0.91, 0.98]	0.81 [0.75, 0.86]
Age + DXA BH femur	0.92 [0.88, 0.96]	0.87 [0.83, 0.92]	0.88 [0.83, 0.93]	0.89 [0.85, 0.93]	0.96 [0.92, 0.99]	0.79 [0.73, 0.85]
Age + DXA BH tibia	0.93 [0.9, 0.97]	0.89 [0.85, 0.93]	0.91 [0.87, 0.96]	0.9 [0.86, 0.93]	0.96 [0.93, 0.99]	0.82 [0.77, 0.87]
FRAX hip	0.8 [0.71, 0.88]	0.74 [0.68, 0.81]	0.73 [0.62, 0.85]	0.75 [0.69, 0.81]	0.89 [0.85, 0.94]	0.69 [0.62, 0.77]
FRAX major	0.78 [0.69, 0.86]	0.75 [0.69, 0.81]	0.74 [0.63, 0.85]	0.76 [0.7, 0.82]	0.89 [0.84, 0.93]	0.71 [0.64, 0.78]
Female						
Age	0.72 [0.63, 0.81]	0.64 [0.53, 0.74]	0.7 [0.59, 0.81]	0.65 [0.54, 0.75]	0.73 [0.63, 0.83]	0.63 [0.53, 0.73]
Age + ibex BH femur	0.88 [0.82, 0.94]	0.79 [0.71, 0.87]	0.83 [0.75, 0.9]	0.8 [0.72, 0.89]	0.89 [0.82, 0.96]	0.74 [0.65, 0.83]
Age + ibex BH tibia	0.91 [0.86, 0.96]	0.88 [0.82, 0.94]	0.9 [0.82, 0.97]	0.87 [0.82, 0.93]	0.91 [0.85, 0.96]	0.83 [0.76, 0.9]
Age + DXA BH femur	0.91 [0.86, 0.97]	0.86 [0.8, 0.93]	0.86 [0.78, 0.94]	0.88 [0.82, 0.94]	0.94 [0.89, 0.98]	0.79 [0.71, 0.87]
Age + DXA BH tibia	0.94 [0.9, 0.98]	0.89 [0.85, 0.93]	0.92 [0.86, 0.97]	0.9 [0.85, 0.95]	0.94 [0.89, 0.99]	0.82 [0.75, 0.89]
FRAX hip	0.78 [0.7, 0.87]	0.71 [0.61, 0.81]	0.7 [0.59, 0.81]	0.72 [0.62, 0.81]	0.78 [0.69, 0.87]	0.72 [0.63, 0.81]
FRAX major	0.76 [0.67, 0.85]	0.73 [0.63, 0.82]	0.75 [0.65, 0.86]	0.74 [0.65, 0.83]	0.75 [0.66, 0.85]	0.73 [0.64, 0.82]
Male						
Age	0.69 [0.5, 0.88]	0.73 [0.63, 0.82]	0.69 [0.5, 0.88]	0.72 [0.63, 0.82]	NA	0.61 [0.48, 0.73]
Age + ibex BH femur	0.78 [0.65, 0.92]	0.89 [0.83, 0.95]	0.83 [0.75, 0.9]	0.89 [0.82, 0.95]	NA	0.79 [0.7, 0.88]
Age + ibex BH tibia	0.87 [0.78, 0.96]	0.88 [0.83, 0.94]	0.87 [0.79, 0.96]	0.87 [0.81, 0.94]	NA	0.77 [0.67, 0.86]
Age + DXA BH femur	0.91 [0.85, 0.96]	0.87 [0.81, 0.94]	0.91 [0.85, 0.97]	0.89 [0.83, 0.95]	NA	0.75 [0.64, 0.85]
Age + DXA BH tibia	0.9 [0.83, 0.98]	0.89 [0.85, 0.93]	0.91 [0.83, 0.98]	0.87 [0.81, 0.94]	NA	0.79 [0.7, 0.88]
FRAX hip	0.7 [0.5, 0.9]	0.73 [0.63, 0.82]	0.7 [0.5, 0.91]	0.75 [0.66, 0.84]	NA	0.6 [0.48, 0.73]
FRAX major	0.69 [0.48, 0.89]	0.73 [0.63, 0.82]	0.69 [0.48, 0.89]	0.74 [0.64, 0.83]	NA	0.6 [0.48, 0.72]

For the test cohort, the NOGG intervention guidelines for FRAX without aBMD as a tool to identify patients with central osteoporosis has a sensitivity of 0.66 (95% CI [0.50, 0.79]) and specificity 0.69 (95% CI [0.63, 0.75]). Using IBEX BH T-score at the PT and an example intervention threshold chosen to achieve comparable sensitivity to FRAX without aBMD, the sensitivity is 0.66 (95% CI [0.50, 0.78]) and specificity 0.94 (95% CI [0.89, 0.96]). If an intervention threshold is chosen to be comparable to the specificity of FRAX without aBMD, the sensitivity is 0.97 (95% CI [0.86, 1.00]) and the specificity is 0.69 95% CI [0.62, 0.75].

When integrated with FRAX clinical risk factors (CRFs) to predict treatment outcome, knee aBMD T-scores enabled more accurate classification of participants that would be recommended treatment after FRAX with aBMD. The AUC for FRAX without aBMD for predicting NOGG treatment recommendation of FRAX with aBMD is 0.80 (95% CI [0.73, 0.86]). With the addition of IBEX BH T-scores to CRFs the AUC was 0.95 (95% CI [0.91, 0.99]) and 0.92 (95% CI [0.87, 0.98]) for the PT and DF ROIs respectively. For DXA, these AUCs are 0.95 (95% CI [0.91, 0.99]) and 0.95 (95% CI [0.92, 0.98]) respectively.

## Discussion

This study supports the clinical validity of opportunistic assessment of bone health from knee radiographs, demonstrating that aBMD estimates derived from the PT and DF closely align with comparable PT and DF DXA results, as well as central DXA results and treatment decisions based on FRAX.

The diagnostic accuracy achieved for osteoporosis prediction and for NOGG-based treatment recommendation mirrors the performance previously observed in forearm radiograph analysis using IBEX BH software (the OFFER1 study), indicating generalizability of this methodology to the lower limb (Lopez et al., 2024; Meertens et al., 2024).

For the healthy-normal cohort in Table 1, the mean T-score for NoF and LS are close to zero and the standard deviation is close to 1. This indicates that the sampled cohort represents the healthy-normal population used for NOF and lumber spine normative data well. As expected, the aBMD and T-scores are on average higher and have smaller standard deviations in the healthy-normal cohort compared to the intended use cohort. Compared to the previous study of IBEX BH applied to wrists (Meertens et al., 2024), the average age of the intended use cohort is lower which may explain why a lower percentage of that cohort have osteoporosis and/or meet the FRAX with NoF aBMD treatment threshold in Table 2. It was noted in the previous study that the average age was above expectations for the intended use cohort.

In Table 3, there is strong correlation for both ROIs but the correlation at the tibia is statistically significantly higher. The standard deviation of the difference is similar for both PT and DF indicating the difference in correlation is partly explained by differences in population standard deviation. Similarly, there is higher correlation for the intended use cohort but the standard deviation of the differences are similar. There is no statistically significant difference between the PT and DF in AUCs for predicting DXA knee T-score ≤ −2.5 or T-score ≤ -1. Having two ROIs available increases the number of processable scans as a valid aBMD and T-score can be returned if one ROI is out of the field of view, fractured or has a surgical implant *in situ*. The sex specific analysis shows some evidence of a better match between DXA and IBEX BH for females, with statistically significant difference in mean and SD of the residuals.

In Table 4, there is no statistically significant difference between IBEX BH and DXA for any of the anatomical sites compared (Central, NoF, LS osteoporosis or osteopenia). However, the IBEX BH DF measures do appear to be performing less well over all measures. Combined with the lower correlation in Table 3, there is evidence to suggest IBEX BH has a higher error, which warrants further research to resolve. One possible explanation is due to the placement of the ROI to avoid the patella; the match between ROIs is not as good at the DF as it is at the PT. The sex specific analysis shows a trend towards better prediction for Females for DXA, IBEX BH and FRAX. However, the effect is not statistically significant so further research would be required to come to a firm conclusion.

The combination of knee T-scores and CRFs appear to improve FRAX without aBMD as a tool to identify patients who would be recommended treatment by NOGG guidelines after FRAX with aBMD. Although FRAX is used as a tool to identify patients who would benefit from treatment, quality and outcomes framework targets recommends not to treat patients for osteoporosis based on FRAX without doing a DXA, even if they have had a fragility fracture, unless they are over 75 or have a vertebral fracture (NHS England, 2024). The National Institute of Health and Care Excellence (UK) also suggests using FRAX for screening for patients that are at high risk of osteoporosis (National Institute of Health and Care Excellence, 2025). Therefore, it is of interest to compare the performance of knee T-scores as a tool to identify patients that would be diagnosed with osteoporosis from a central DXA scan. In Table 4, FRAX without aBMD 10-year fracture risk measures appear to perform worse than T-scores at the knee, and only perform marginally better than age. The superiority of IBEX BH PT T-score is further evidenced by the operating point analysis where, for the same sensitivity, the specificity of IBEX BH is significantly higher than FRAX without aBMD referral threshold, and visa-versa.

Globally, over 8.9 million fractures occur annually due to osteoporosis, with hip and femoral fractures accounting for a disproportionate share of morbidity, mortality, and healthcare utilization. In the UK alone, fragility fractures cost the NHS over £4.4 billion annually, yet up to 50% of at-risk patients remain undiagnosed and untreated (Söreskog et al., 2025). Given the high proportion of knee radiographs in the elderly population, our results suggest an opportunity to identify patients at high risk of fragility fracture, contributing to a reduction in the existing treatment gap for osteoporosis. This is particularly pertinent, as many patients will have knee radiographs prior to knee surgery, where offloading is known to cause bone density loss in the affected limb rapidly over a short period of time post-operatively (Chang et al., 2023; Chang et al., 2022), and a complex relationship between osteoporosis and osteoarthritis is still being unpicked (Hu et al., 2020).

Prior cost-effectiveness analysis demonstrates the economic benefits from integrating IBEX BH into DR pathways, even allowing for flexibility in deployment in terms of target thresholds and restricted demographics (Söreskog et al., 2025). Its ability to function as a “passive” screen, requiring no change to imaging protocols, position it for rapid deployment at scale. The observed specificity of the knee-based risk prediction model when matched for FRAX without aBMD sensitivity demonstrates clear clinical utility. By reducing false positives relative to FRAX alone, this approach may minimize unnecessary DXA scans, enabling more efficient resource use.

The results are contextualised as high performing when compared with osteoporosis screening tools that target the PT, with the advantage of being opportunistic when compared with ultrasound based systems that screen for osteoporosis based on cortical thickness (Gokcek et al., 2023; Karjalainen et al., 2017; Karjalainen et al., 2016). Similarly our results generally perform favourably to other screening systems, such as those utilising quantitative ultrasound (Nayak et al., 2006) or novel ultrasonic techniques applied at PT (Vogl et al., 2019; Armbrecht et al., 2021; Kay et al., 2022).

An advantage of IBEX BH is that it produces a report with well understood biomarkers that are familiar to clinicians. The unique interpretable and auditable methodology behind IBEX BH makes it analogous to DXA as it is based on the unique scattering and attenuating properties of soft tissue and bone. This makes it a suitable future application for referral to DXA, and potentially an alternative with development of a larger clinical evidence base, especially where DXA coverage is sparse. Results also suggest comparable or dominant performance over black box AI models for osteoporosis detection utilising x-ray (He et al., 2018; Xie et al., 2024).

In this study, both central osteopenia and osteoporosis predictions are presented to enable direct comparison with published studies from other screening tools, two of which are deep learning models limited to osteopenia predictions. One compares to central osteopenia (using images of multiple body parts) and one to NoF osteopenia (using hip and pelvis images). For the first approach, the AUCs for three datasets evaluated were 0.89, 0.87 and 0.82 for all body parts and 0.87, 0.86 and 0.76 for the knee (Bilbily et al., 2024) compared to 0.89 (95% CI [0.84, 0.93]) for IBEX BH. For the second, the AUC is 0.83 compared to 0.87 (95% CI [0.83, 0.92]) for IBEX BH (Pignolo et al., 2025). The results presented here compare well with the other methods. It is noteworthy that IBEX BH using knee images to predict NOF osteopenia appears to outperform the deep learning method that uses hip images but a direct study using both methods on the same participants would be needed to draw a firm conclusion. The use of osteopenia as a target classification also has drawbacks. In the intended use cohort, 64% of participants with NoF osteoporosis required treatment by NOGG guidelines for FRAX with aBMD while only 10% of those with osteopenia required treatment. Furthermore, 55% of the over 50 cohort had osteopenia. Therefore, although there may be benefits to identifying patients with osteopenia to incentivise bone health positive lifestyle changes, particularly when DXA resource is scarce, identifying patients with osteopenia could be inefficient and result in too many DXA scans that do not result in a patient receiving treatment.

Public trust is essential for clinical artificial intelligence (AI) adoption. Findings from Manning et al. show that transparency, clinician engagement, and regulatory backing are critical for patient confidence. IBEX BH is not a black box AI method so is inherently more transparent and auditable utilising the underlying physics of X-ray imaging. However, communicating this to clinicians and regulators is critical. Yet qualitative analysis of public perception indicates strong support for opportunistic screening, particularly from those who have experienced the impact of osteoporosis directly (Manning et al., 2023). Evidence also supports that patients will engage with treatment when opportunistically identified, even in the absence of fragility fracture, which is almost always the first indicator of bone density loss (James and Meertens, 2025).

Although the initial use for IBEX BH is for opportunistic screening, there could be applications for its elective use as a tool to measure knee aBMD. Two reasons DXA use on a knee is limited to research are i) there is typically no standard clinical DXA protocol or ROI template for the knee and ii) there is a lack of normative data to contextualise the aBMD values in T-scores. This work goes some way to address this with IBEX BH with a set protocol, ROI template and normative data. A third reason is that the association between knee aBMD and wider skeletal fracture risk is uncertain. However, forearm, LS and NoF aBMD have all been demonstrated to have utility as independent predictors of fracture risk, despite having better predictive capabilities of fracture at the localised anatomical region compared with wider skeletal fracture prediction (Marshall et al., 1996). Therefore, knee could also have independent information when predicting fracture, particularly of the leg. Further research may identify additional uses of knee aBMD provided by IBEX BH, justifying its use as an elective bone densitometer.

Given its intended use as a passive opportunistic screening tool, the IBEX BH report is designed to provide familiar biomarkers to clinicians in a concise and uncluttered way. However, IBEX BH also produces a high-resolution bone density map, see Figure 1. If further clinical value of the density maps is evidenced, it would further justify the use of IEBX BH electively. For example, research into the clinical value of this density map could show further utility in surgical planning or sports science applications. Furthermore, with further development, bone quality (rather than quantity) measures like trabecular bone score (McCloskey et al., 2016) or volumetric aBMD (Silva et al., 2013) could be calculated from the high-resolution map.

## Limitations

There are some limitations of the use of the knee radiograph for opportunistic osteoporosis detection. Whilst indicative of systemic bone density, it is known that skeletal changes are not uniform and may be influenced specifically at the tibia by an absence/abundance of weightbearing exercise in comparison to the hip (Zheng et al., 2021) and radius (Johannesdottir et al., 2022). Similarly, trabecular bone loss (such as at the spine) is accelerated with ageing relative to cortical bone (dominant at the PT and DF) which may be more protected by weight bearing exercise (Demontiero et al., 2012). Future applications for bone health monitoring would need to consider that response to medical therapy may be different across anatomical locations (Choksi et al., 2018).

Although this study was performed at a single centre, a relatively large and diverse reference and target population of participants has been recruited. Further studies are needed to evidence i) the repeatability of the software over time, ii) performance across different software manufacturers and iii) the performance on a wider patient demographic including ethnic minorities. Furthermore, the use of a volunteer population means that it is likely fewer participants exhibited co-morbidities relative to clinical practice. Most pertinently, there were no fractures present in any of the knees analysed. Further, clinically-based studies are needed to evidence that these results can be achieved on the intended use population in clinical practice.

Finally, the ROIs were semi-automated where the automated component required a segmentation of the femur, tibia and patella. This segmentation will have to be automated before the method can be put into clinical use.

## Conclusion

The findings of this study extend the utility of IBEX BH opportunistic osteoporosis screening to routine knee radiographs, offering a novel approach to identify patients at increased risk of fragility fracture via analysis of the PT and DF. When integrated into routine workflows, this strategy has the potential to address longstanding diagnostic and treatment gaps in osteoporosis care, improving patient outcomes while reducing healthcare system costs. Further research is encouraged in a clinical setting.

## Data Availability

The raw data supporting the conclusions of this article will be made available by the authors, without undue reservation.

## Abbreviations

AP, Anterior Posterior; aBMD, areal bone mineral density; CRFs, clinical risk factors; CI, confidence interval; DXA, Dual-energy X-ray Absorptiometry; DR, Digital Radiograph; DICOM, Digital Imaging and Communications in Medicine; DF, Distal Femur; FRAX, Fracture Risk Assessment Tool; IBEX BH, IBEX Bone Health; LS, lumbar spine; NoF, Neck of Femur; NOGG, National Osteoporosis Guideline Group; PT, Proximal Tibia; ROI, Region of Interest; QUS, quantitative ultrasound; SID, source to image distance.
